# Difference between Okinawan and Dutch older adults in prefrontal brain activation

**DOI:** 10.3389/fnagi.2025.1454068

**Published:** 2025-07-31

**Authors:** Branislava Ćurčić-Blake, Yuko Futenma, Donald Craig Willcox, Parisa Esmaeili Tazangi, Nido Dipo Wardana, Yukihiko Ueda, André Aleman

**Affiliations:** ^1^Cognitive Neuroscience Center, Department of Biomedical Science of Cells and Systems, University of Groningen, University Medical Center Groningen, Groningen, Netherlands; ^2^Graduate School of Regional Culture, Okinawa International University, Okinawa, Japan; ^3^Department of Human Welfare, Okinawa International University, Okinawa, Japan; ^4^Department of Research, Kuakini Medical Center, Honolulu, HI, United States; ^5^Faculty of Psychology, Universitas Airlangga, Surabaya, Indonesia; ^6^Faculty of Psychology and Neuroscience, Maastricht University, Maastricht, Netherlands

**Keywords:** healthy aging, Okinawa, blue zones, functional near infrared spectroscopy (fNIRS), executive function, prefrontal brain activation

## Abstract

**Background:**

Older adults in Okinawa (Japan) are known for healthy aging and longevity. This is the first study to explore brain activation during executive functioning in Okinawan older adults in comparison to Western-European (Dutch) older adults.

**Methods:**

A total number of 80 participants were included in the study (41 from the Netherlands and 39 from Okinawa), with ages between 65 and 80 years). The groups did not differ for sex and handedness. Brain activation was measured during a visual working memory task and a verbal fluency task, for bilateral frontal cortex using functional near infrared spectroscopy (fNIRS). We investigated oxygenated hemoglobin (HbO) levels and laterality index.

**Results:**

Both groups performed within the normal range for their population. During verbal fluency, less activation in the left frontal gyrus was observed in Okinawa participants as compared to Dutch participants, and more activation in the anterior superior parts of the frontal gyrus. For the n-back task, the Okinawa group exhibited less activation in the right dorsolateral prefrontal cortex and more activation in the bilateral anterior frontal gyrus. Although laterality indices were similar for both tasks, Okinawa participants showed stronger left lateralization during category fluency.

**Conclusion:**

Our results reveal less activation of the task-relevant areas in participants from Okinawa as compared to Dutch participants. It could be hypothesized, with caution, that Okinawan older adults may need less executive processing resources to perform the task. Other differences in activation may be related to different strategy use, which may be studied in more detail in future investigations.

## Introduction

Aging is associated with a decline of specific cognitive functions, also in individuals without neurological or psychiatric disorders. Even though some functions, such as language and crystallized intelligence are resistant to the effect of aging, other important domains are affected. Specifically, functions such as memory and executive functioning (e.g., planning, mental flexibility, inhibition and working memory updating) have consistently been shown to decline with age (Ardila et al., 2000). Older adults from Okinawa (Japan) have been reported to be in better health in comparison to older adults in Western Europe, North America, or even to mainland Japan (Poulain and Herm, 2024; Willcox and Willcox, 2014). That is, Okinawan older adults suffer significantly less from age-related diseases, including neurodegenerative disorders such as senile dementia (Ogura et al., 1995). Okinawa also counts with a disproportionate number of centenarians (Poulain and Herm, 2024; Willcox et al., 2017). This can be attributed in small part to genetic differences and to a larger extent to environmental variables, such as diet (Willcox et al., 2017, Gavrilova and Gavrilov, 2011)

However, there is a lack of research studying functional brain characteristics of successful aging as found in Okinawan older adults. The current study was designed to take the first step in this regard. Specifically, we wanted to investigate brain activation during executive functioning in Okinawan older adults as compared to Western-European older adults (in this case, Dutch older adults).

Prefrontal brain regions are important for many higher order cognitive processes including episodic memory and executive function (Simard et al., 2003). They are part of the fronto-temporo-parietal network (Niendam et al., 2012) that is involved in cognitive control and central executive function (Seeley et al., 2007, Zanto and Gazzaley, 2013). Importantly, measurement of activation of the prefrontal cortex (PFC) is feasible with functional near infrared spectroscopy (fNIRS). fNIRS is a method that measures changes in oxygenated and de-oxygenated hemoglobin (HbO and Hb) from the cortex, which are proportional to the activation of underlying neural tissue. This is similar to blood-oxygen-level-dependent (BOLD) measurement by functional MRI. fNIRS is easy to apply, and compared to fMRI, can be used in more natural and comfortable conditions (compared to lying in a noisy MRI scanner). Furthermore, fNIRS is considered a convenient technique for studying brain activation in older adults (Doherty et al., 2023) due to its comfort and ease of use, particularly in the frontal areas. This is especially important given the challenges associated with measuring brain activity in this population, such as hearing impairments (Getzmann et al., 2023).In order to assess functioning of the PFC, we chose verbal fluency and non-verbal working memory. These two tasks were chosen as we wanted to assess functions relying crucially either on the left hemisphere (verbal fluency) or the right hemisphere (non-verbal working memory). In addition, a prerequisite for the tasks was that they should be feasible for measurement with fNIRS as shown by previous studies. Verbal fluency is an executive function task requiring coordinated activity of the frontal and temporal lobes (Wagner et al., 2014). PFC function during a verbal fluency task has been investigated in older Japanese people (Pu et al., 2014) and in mild cognitive impairment (MCI) patients (Yeung et al., 2016). Pu et al. (2014) demonstrated that fNIRS can be applied successfully in older adults during the verbal fluency task. Yeung et al. (2016) showed that older adults with normal cognition still preserve the left lateralization of frontal activation during the verbal fluency task, whereas MCI patients have significantly less lateralization. Given the overall health advantages of Okinawan elderly in comparison to Western European elderly, we expected Okinawan elderly to have more preserved brain functioning. Therefore, we predicted that the Western European older adults would show more neural signs of cognitive decline, i.e., in the direction of the pattern observed with MCI, whereas the Okinawan older adults may have a more “youthful pattern.” This could be apparent by less activation during accurate performance of the task and stronger lateralization toward the left hemisphere during verbal fluency (Yeung et al., 2016). Factors associated with a healthy lifestyle have been shown to be associated with delayed brain aging and a reduced risk of cognitive decline. For example, a large-scale study found health benefits in individuals engaging in five healthy lifestyle habits—non-smoking, physical activity, light to moderate alcohol consumption, MIND diet, and engagement in late-life cognitive activities (Dhana et al., 2020). Specifically, compared to those with 0–1 healthy lifestyle factor, the risk of Alzheimer dementia was 60% lower in those with 4–5 healthy lifestyle factors. Notably, most of these factors, especially healthy diet, regular exercise and late-life cognitive activities, are characteristic of Okinawan elderly (interestingly, there is no word for retirement in Okinawan language). Regarding diet, the MIND diet—a blend of the Mediterranean and DASH diets, similar to the Okinawan diet—has been linked to slower cognitive decline. Morris et al. (2015) reported that high adherence to the MIND diet reduced the risk of Alzheimer’s disease substantially. Together, these findings underscore that a healthy lifestyle impacts on maintaining brain health well into older age. Franz et al. (2021) investigated the influence of lifestyle on brain aging after nearly 30 years. Using a lifestyle behavior composite based on data collected at age 40, the participants were measured with MRI brain scans at mean age 68. The results showed that having more favorable lifestyle behaviors predicted less advanced brain age and less AD-like brain aging.

Another aim was to investigate working memory functioning in the same groups, given its sensitivity to aging and reliance on PFC networks. Working memory, in addition to episodic memory and processing speed, belongs to the cognitive functions most affected by aging (Aleman, 2015). Memory functioning has also been investigated in older adults using fNIRS during memory encoding (Ferreri et al., 2014) and memory retrieval (Obayashi and Hara, 2013). One Japanese study demonstrated that the right PFC plays an important role for the correct retrieval of memorized meaningless shapes (Sanefuji et al., 2007). Such a task is particularly convenient to compare European and Japanese older adults, as it is not affected by issues such as phonetic differences of the two languages. In a study using fNIRS with a visuospatial working memory task, Lee et al. (2023) showed that older adults exhibited higher activation than younger adults under more difficult but not easier levels. They also reported that older adults whose performance was comparable with that of young adults showed more right-lateralized activation. Translating this to predictions regarding Okinawan and Dutch elderly, we would expect higher activation in the Dutch elderly (with equal performance), as the Okinawan participants are expected to show a more youthful pattern of brain activation and hence need less neural resources.

We limited our investigations to two tasks during fNIRS because Ikezawa et al. (2009) showed that a lower number of tasks and shorter task duration are better for clinical examinations as prolonged fNIRS measurements yielded decreases in HbO activation.

In summary, we predicted that Okinawan older adults would need less task-specific brain activation (neural resources) compared to Dutch older adults to perform the tasks. We hypothesized this to be manifest primarily in the left PFC for the verbal fluency task and in the right PFC for the visual working memory task, with stronger lateralization in the Okinawan participants.

## Materials and methods

### Participants

The study involved 41 participants from Groningen, the Netherlands, and 39 from Okinawa, Japan. Inclusion criteria comprised ages 65–80, Dutch fluency for Groningen participants, and Japanese fluency for Okinawan participants. We were aware that Japanese participant would produce less words on verbal fluency than their Dutch counterparts, because of structural language differences (Itou, 2006). However, this does not have to affect the comparability of neural activation, because participants from both groups engage in the same task during the short time given by trying to produce words. Exclusion criteria included current psychiatric/neurological diagnoses, Mini Mental State examination (MMSE) scores below 27, and alcohol or drug abuse. Attempts were made to include participants of similar age, sex and education across groups, by actively searching for participants with similar characteristics.

### Ethics

All study protocols were fully approved by the medical ethical board of the University Medical Center Groningen (METC; UMCG reference number METc2018.368) and the research ethical board of the Okinawa International University (reference number 98). All procedures were carried out according to the declaration of Helsinki and registered in the UMCG Research Registry Number 201800495.

Participants in the study signed informed consent, and only those fully capable of deciding to participate in the study were included. No follow-up was conducted after the study.

### Procedure

Participants were recruited via advertisements and word of mouth. In Groningen, information was also sent to participants who could not participate in a previous study on executive functioning in elderly people but had indicated that they would like to receive information about this new study. Participants first underwent neuropsychological tests and, if meeting inclusion criteria, engaged in in the further procedure. The data collection was in both places performed between September and March the next year. The same fNIRS device was used both for Okinawa and Groningen. The study was set up at corresponding labs of each institute. fNIRS measurements during a verbal fluency task (involving letter fluency and category were done fluency) and a visual n-back task (for more details, see Supplementary materials). The participants were seated comfortably, the fNIRS cap and optodes were placed, and non-verbal communication was agreed upon, with the operator to prevent interference with language processing areas, as fNIRS sites overlapped.

#### Tasks

##### Verbal fluency

In this test subjects were given one minute to produce as many words as possible belonging to a given category. In 2 semantic fluency conditions, they had to name animals and occupations. In 2 phonological fluency conditions, they had to name words beginning with the letter K and A (in Dutch). In Okinawa, a validated Japanese version was used. The verbal fluency test is a highly sensitive indicator of general cognitive decline (Lezak et al., 2004). In terms of executive functions, it requires monitoring the words already produced and organizing search in semantic or phonological memory.

##### N-back task

**FIGURE 1:**
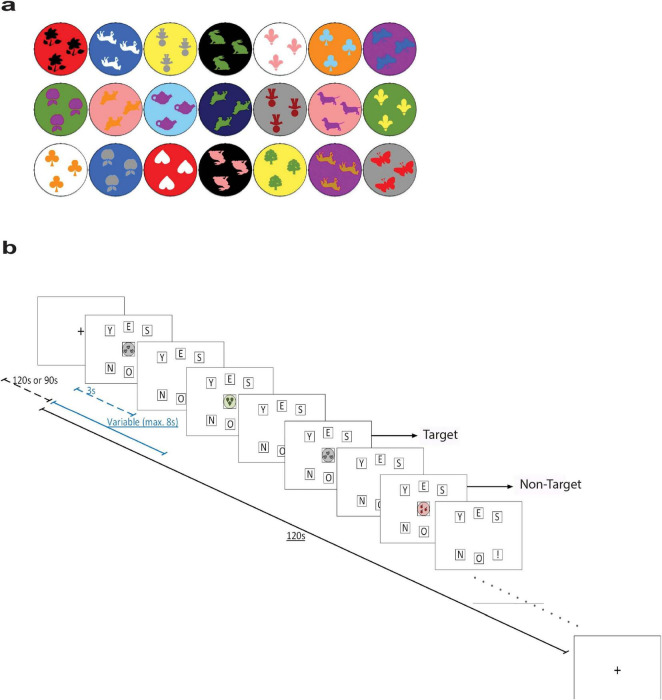
**(a)** The stimuli shapes presented in the n-back task. **(b)** Schematic illustration of the n-back task. Dashed blue line represents the duration of stimulus presentation (3 s), while the solid blue line represents the duration of one trial (variable with a maximum duration of 8 s). Dashed black line represents the duration of the baseline period (120 or 90 s), while the solid black line represents the duration of one block (120 s). Pictures are based on the Presentation software (Neurobehavioral Systems www.neuorbs.com), adapted from Kaufman (2004).

The n-back task stimuli were displayed individually and centered on the monitor. We employed a 2-back task with 21 shapes (Figure 1). Responses were recorded by button press, with three buttons (“Y,” “E,” “S”) and another three buttons below them (“N,” “O,” and “!”). Participants were instructed to press “Y,” “E,” and “S” if the stimulus on screen was the same as the one shown two times back (i.e., target stimulus), otherwise the bottom row buttons (denoting NO!) had to be pressed (i.e., non-target stimulus). Each stimulus was presented for 3 s and followed by 8 s of response duration. The next stimulus was to be presented after participants completed their response or if the response duration was up. The task lasted for 120 s and was designed such that target stimuli were presented 50% of the time.

Every participant was given at least one practice trial with auditory feedback signaling when they made a correct or incorrect response. Then, this was followed by three assessment trials during which brain activity was recorded, and the auditory feedback was omitted. At the beginning and the end of the recording session, participants were asked to look at a yellow fixation cross situated at the center of the monitor for 120 s. The fixation cross was also presented during a 90 s break interval between assessment trials. The schematic diagram of the n-back task is presented in Figure 1.

#### Additional measures

A number of tests and questionnaires were used to characterize the groups in terms of cognitive status, social functioning and physical activity. The neuropsychological tests included: (a) Digit Span is a subtest included in both the Wechsler Memory Scale-III (WSM-III) and the Wechsler Adult Intelligence Scale (WAIS) and consists of two versions (Griffin and Heffernan, 1983). The Digits Forward (Forwards) is considered as a simple memory span test, while the Digits Backward (Backwards) is a more complex test with an executive component (Wilde et al., 2004); (b) Symbol Digit Modalities Test (SDMT) was originally developed to identify neuropsychological impairments and assesses divided attention, visual scanning, and motor speed (Sheridan et al., 2006; Strauss et al., 2006); (c) Trail Making Test (TMT) consists of two parts where part A (TMT-A) assesses attention and speed, while part B (TMT-B) also involves divided attention and mental flexibility; (d) Stroop Test was initially developed by Stroop (Stroop, 1935) and is a measure of selective attention and cognitive flexibility; and (e) Mini Mental State Examination (MMSE) was developed by Folstein et al. (1975) and has been a popular measure to screen cognitive impairments especially in older adults (Folstein et al., 1975; Strauss et al., 2006). In addition, we administered other screening measures, including (f) Edinburgh Handedness Inventory, a 10-item questionnaire assessing handedness which was developed by Oldfield (Oldfield, 1971); (g) Social Functioning Scale (SFS) assesses seven areas that are crucial for social competence, namely social engagement/withdrawal, interpersonal behavior, prosocial activities, recreation, competence for independence, the performance of independence, and employment or occupation (Birchwood et al., 1990); and (h) Physical Activity Scale for the Elderly (PASE) assesses physical activities commonly engaged in by older persons (Washburn et al., 1993).

### NIRS acquisition

**FIGURE 2:**
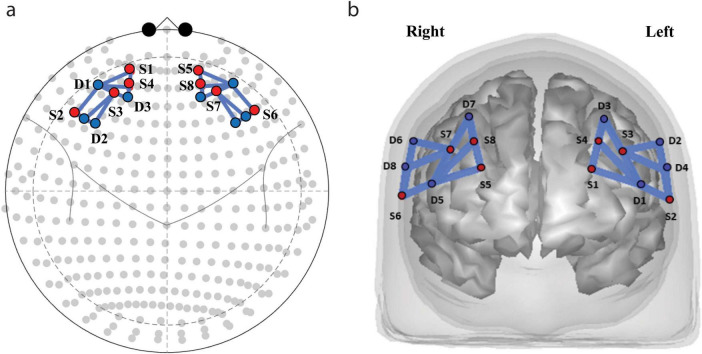
Functional near infrared spectroscopy (fNIRS) sources and detectors locations. Optodes (S, sources; D, detectors) and channel placement. **(a)** Placement of the sources and detectors on the cap. **(b)** Distribution of sources (red dots) and detectors (blue dots) projected on the brain surface.

Concentration changes of oxygenated (HbO) and deoxygenated (Hb) hemoglobin were recorded by NIRSsport (Germany, United States^1^) using eight source and eight detector optodes at two wavelengths (760 and 830 nm) and a sampling frequency of 7.8125 Hz. Optodes were distributed equally on the left and the right hemisphere over the PFC and frontal cortex area in accordance with the International 10/20 EEG system (see Supplementary Table 1). Altogether, we used 22 source-detector pairs or channels. Figure 2 depicts the source-detector arrangements. The list of channels, optodes and the distances is presented in the Supplementary Table 2.

### NIRS data analysis

We estimated levels of Oxygenated hemoglobin (HbO) using the AnalyzIR Toolbox (Santosa et al., 2018) and Matlab 2019b (TheMathworks, Natick, MA, United States). First, the raw fNIRS data of the tasks input into the AnalyzIRToolbox. Data were inspected for quality using ta coefficient of variance (CV) greater than 7.5% for either wavelength were excluded from the analysis. CV indicates the signal-to-noise ratio (SNR), with lower values implying better SNR and therefore can be used to control for the data quality. It is calculated from the absorption data of each measurement wavelength using the following formula: $C\,V=100\times \frac{S\,t\,a\,n\,d\,a\,r\,d\,D\,e\,v\,i\,a\,t\,i\,o\,n}{M\,e\,a\,n}$. To correct for motion artifacts, which occur when participant moves, and is visible as a transient change in levels of Hb often larger than brain activation, Principal Component Analysis (PCA) and wavelet analysis has been applied (Santosa et al., 2018). PCA detect the common motion artifacts across channels and the wavelet analysis is used to filter those. Then, in order to remove other physiological artifacts (e.g., slow drifts, respiration), a band-pass filter was applied to the absorption data. A low-frequency cut-off of 0.01 Hz and a high-frequency cut-off of 0.2 Hz were used (Zhang et al., 2017).

Finally, the absorption data were converted into concentration data using the modified Beer-Lambert (for details see Methods section in Supplementary materials, the Conversion from optical density to Hb and HbO levels Subsection) After the data preprocessing, the data were visually inspected. Three participants from Groningen were excluded from the analysis of the of n-back task [two because of noisy data and one because of extensive motion (subject no 15)]. Regarding verbal fluency, two participants were excluded from the analysis [one from Okinawa because problems with the task execution and one from Groningen because of problems with noisy data (subject no 15)]. Note that subject no 15 was excluded from both tasks, so he was fully excluded from further analysis (including behavioral data and demographics).

General Linear Model (GLM) was applied in a block design. We analyzed HbO levels as the oxygenated hemoglobin signal is stronger and more reliable than the deoxygenated hemoglobin signal, and many studies have focused on studying this signal. Short distance channels [with the distance less than 1.5 cm (Supplementary Table 2)] were used as covariate.

To compare the brain activation level and laterality index between the two groups, firstly, the first-level statistical analysis was conducted using the NIRS-SPM approach to estimate activation during task performance. A discrete cosine transform (DCT) with a high pass filter of 0.008 Hz (i.e., disregarding signals that fluctuate in periods longer than 128 s) and a Gaussian pre-coloring method to account for the serially-correlated noise in the advanced general linear model were used. The beta values from this analysis were used as estimates of the brain activity levels for each channel. The beta values were then utilized for group comparison (second-level analysis). The group comparison was then continued for each task separately, using beta values for each fNIRS channel. To investigate the brain activation, a Linear Mixed Effect (LME) Model per channel, was fitted in MATLAB R2019b, with a random intercept for each participant, and fixed effect including Group [Brain Activation ∼ Group + (1| Participant)]. A contrast was then applied to the model to test the statistical significance of the difference between two groups. To correct for multiple comparisons due to multiple fNIRS channels, *p*-values were adjusted using the false discovery rate (FDR) correction.

Due to differences in educational systems (see Supplementary materials, Supplementary Table 4 subsection Education Level Adjustment for details), we decided before analysis to repeat the analyses while controlling for education.

### Time courses

A grand averaging was conducted using HOMER analysis toolbox (Huppert et al., 2009) to visualize the change of HbO levels over time. The concentration level within a second before the start of trials was used as the baseline level. The pattern of hemodynamic response would be visually inspected. Average time courses were calculated per participant per time point from 1 s before the onset of the stimulation burst to 60 s after.

### Laterality index

We also hypothesized that the Okinawa group would demonstrate more left lateralization, as shown by a comparison of the laterality indices (LIs) of the two groups. LI can be calculated using the following formula for each subject: LI = (AL–AR) / (| AL| +| AR|) where AL is the average beta value of left hemisphere channels, and AR is the mean of beta values of right hemisphere channels. We first computed the average beta values per hemisphere and per subject. Then, the LI coefficient was calculated per subject. In addition, because the verbal fluency task activates mainly lateral parts of the frontal cortex, we repeated this procedure only including channels in the lateral part of the frontal cortex.

#### Statistics

Before analyzing the data, normality was checked through Shapiro–Wilk tests. Subsequently, any non-normal data were log-transformed for suitability for parametric testing. If the log-transformed variables still violated the normality assumption, then non-parametric tests were conducted. The data screening and analysis were performed using IBM SPSS Statistics 25 (IBM Corp., Armonk, NY, United States). Effect sizes where relevant, were calculated using online-tool: https://www.psychometrica.de/effect_size.html. The FDR correction for multiple comparisons was used to correct for multiple comparisons.

## Results

### Demographics

The two groups were matched for sex (Table 1). There was difference in age of 2.9 years which was statistically significant. However, all participant were between 64 and 80 (Groningen participants were up to 77 years old), thus belonging to the same age range. Educational level (according to the classification in Supplementary Table 3) did not differ significantly between groups. In addition, The groups did not differ in cognitive or social functioning measures (i.e., MMSE and SFS) but, as expected, there was a difference in the physical activities as measured by PASE. Given the difference in age, PASE scores and the presence of three left-handed subjects in Dutch group, we repeated subsequent HbO analyses excluding three left-handed participants and subsequently adding age and PASE as covariates.

**TABLE 1:** Descriptive statistics for the samples, including cognitive test performance, SFS and PASE.

Variable	Mean (SD)		Significance (*p*-value)
	Groningen (*n* = 38)	Okinawa (*n* = 37)	Groningen vs. Okinawa
Age	69.81 (3.66)	72.7 (4.35)	t (73) = −3.11 (0.003)
Education level (high/medium/low)	35/1/2	32/0/5	χ(2,75) = 2.41 (0.30)
Gender (female)	20	23	χ(1,75) = 0.70 (0.40)
Handedness	75.72 (26.6)	89.79 (10.12)	t (47.7) = −3.04 (0.004)
**Digit span**			
Forward^a^	6.21 (2.04)	8.92 (1.63)	t (73) = −6.32 (< 0.001)
Backward	6.39 (1.99)	5.54 (1.85)	t (73) = 1.92 (0.06)
SDMT	47 (6.76)	42.05 (9.38)	t (65.4) = 2.61 (0.01)
**TMT**			
TMT-A^a^	32.32 (8.56)	125.22 (39.06)	U = 0 (< 0.001)
TMT-B^a^	71.55 (23.62)	150.76 (54.14)	U = 52.5 (< 0.001)
**Stroop test**			
Stroop I – time^a^	48.37 (8.09)	28.65 (6.33)	U = 30.5 (< 0.001)
Stroop I – correct^b^	99.84 (0.49)	47.95 (0.22)	U = 0 ( < 0.001)
Stroop II – time^a^	59.92 (9.57)	29.78 (6.59)	U = 3 (< 0.001)
Stroop II – correct^b^	99.66 (0.66)	47.51 (1.38)	U = 0 (< 0.001)
Stroop III – time^a^	97.05 (19.14)	52.49 (16.25)	U = 45.5 (< 0.001)
Stroop III – correct^b^	99.13 (2.94)	46.92 (1.83)	U = 0 (< 0.001)
MMSE	29.47 (1.79)	28.78 (1.1)	U = 541 (0.07)
SFS	160.84 (14.32)	157.89 (13.49)	t (73) = 0.92 (0.36)
PASE	171.99 (63.63)	139.22 (48.75)	U = 476 (0.02)

^a^ Not comparable due to the use of different versions of the tests. ^b^Proportion correct is used here due to the use of different versions of the test. ^c^50th percentile. ^d^Normalized range for the average age of the group. Descriptive statistics for the groups included. The left column lists the demographic variables, cognitive test performance, SFS and PASE. The second and third columns from the left show average values of the variables across the group, with their standard deviations in brackets. For classification of Education level see section in Methods. Welch’s *t*-test was used for handedness, and SDMT. Non-parametric test was used to test the group difference for sex and education level (Chi-square) as well as TMT, Stroop task, MMSE, and PASE (Mann-Whitney U test). SDMT, Symbol Digit Modalities Test; TMT, Trail-Making Task; MMSE, Mini Mental State Examination; SFS, Social Functioning Scale; PASE, Physical Activity Scale for the Elderly.

The Okinawan participants produced less words than the Dutch participants on the verbal fluency tasks (letter 16.5 ± 5.4 and 26.2 ± 7.5, category 25.9 ± 4.4 and 40.6 ± 9.2 for Okinawa and Dutch groups, respectively; Supplementary Table 5a), as expected due to language differences. Notably, scores of both groups in category fluency fall within the normal range [z-values ∈(−2,2)] but Okinawan participants are performing better, as expected [according to higher z-values (Supplementary Table 5b)].

The two groups did not differ in accuracy (Supplementary Table 6) for the n-back task (U = 559, *p* = .12, Cohen’s d = .358). Z-values for cognitive performance, where available, are summarized in the Supplementary Table 7.

### fNIRS activation results

#### Verbal fluency task

**FIGURE 3:**
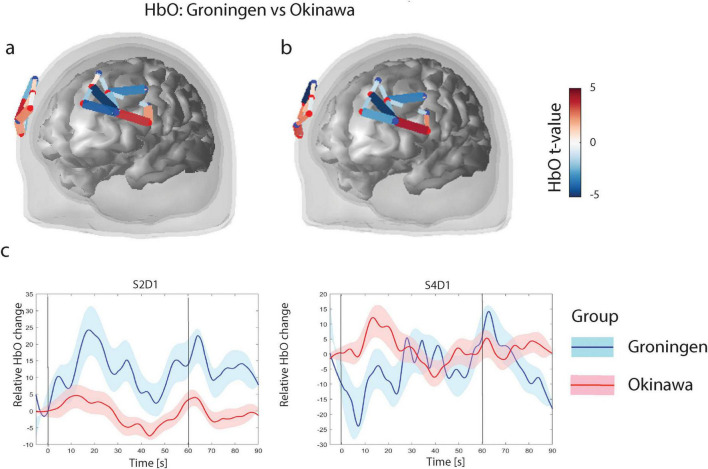
**(a)** Verbal fluency level contrasts for oxygenised hemoglobin (HbO). Only significant channels (that survive FDR corrected threshold *p*_*FDR*_ < 0.05) are shown. The color scale on the right represents the t-value of the channels indicated in the figures under **(a,b)**. For HbO contrasts, positive t-values (red) correspond to relatively larger activity in the Groningen group as compared to Okinawa group, and negative t-values (blue) correspond to larger activity for the Okinawa group. **(b)** Verbal fluency HbO level contrasts between groups. Same as **(a)** after correction for educational level. **(c)** Relative levels of HbO time courses. Level of Oxyhemoglobin in two different channels for two groups. Black vertical line denotes onset (at t = 0 s) and offset of the trial (at t = 60 s). Solid lines represent grand averages of time courses and shaded areas represent standard error of mean (SEM): red, Okinawa group; blue, Groningen group.

During the verbal fluency task, the Groningen group had higher (*p*_FDR_ < 0.05) HbO levels, compared to the Okinawa group, in the rostral part of the superior and middle frontal gyrus (right hemisphere; three channels) and in the middle frontal (rostral part, left hemisphere; one channel). Furthermore, the Groningen group showed lower HbO levels than the Okinawa group in the caudal part of the superior and rostral part of the inferior frontal gyrus (right hemisphere; two channels) and in the left rostral part of the superior frontal gyrus (two channels) besides the middle frontal gyrus (caudal part; one channel), c.f. Figure 3.

After correction for educational level, results in the right hemisphere remained largely the same and in the left hemisphere the significance levels were slightly lower (Figure 3b). The time courses of the representative channels are presented in Figure 3c. The Groningen group exhibits a sharper initial increase in HbO levels, followed by sustained activation. In contrast, the Okinawa group displays a more gradual rise with lower peak levels. The results remained consistent after repeating the analysis without the three left-handed participants and adding age and PASE as covariates (Supplementary Figure 2).

#### n-back task

**FIGURE 4:**
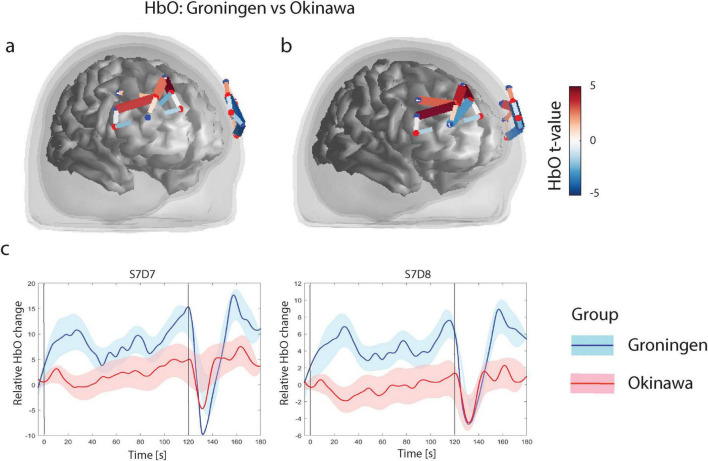
**(a)** N-back level contrasts for oxygenised hemoglobin (HbO). Only significant channels (that survive FDR corrected threshold *p*_*FDR*_ < 0.05) are shown. The color scale on the right represents the t-value of the channels indicated in the figures under **(a,b)**. For HbO contrasts, positive t-values (red) correspond to relatively larger activity in Groningen group as compared to Okinawa group, and negative t-values (blue) correspond to larger activity for the s Okinawa group. **(b)** N-back level contrasts for HbO, after correction for educational level. **(c)** Relative HbO levels time courses. Level of Oxyhemoglobin in two different channels for two groups. Black vertical line denotes onset (at t = 0 s) and offset of the trial (at t = 60 s). Solid lines represent grand averages of time courses and shaded areas represent standard error of mean (SEM): red, Okinawa group; blue, Groningen group.

The GLM analysis revealed three channels in the superior and middle frontal gyrus in the right hemisphere and two channels in caudal part of the middle frontal gyrus in the left hemisphere where Groningen group had significantly larger HbO levels (*p*_*FDR*_ < 0.05) relative to the Okinawa group. Further, in the rostral part of the superior and middle frontal gyrus bilaterally Groningen group had lower HbO levels relative to the Okinawa group (*p*_*FDR*_ < 0.05), Figure 4.

After correction for educational level, the results remained largely the same (see Figure 4b). The time courses of the representative channels are presented in Figure 4c. The Groningen group demonstrates a more pronounced and sustained HbO increase compared to the Okinawa group, which shows a slower rise and earlier return to baseline. The results remained consistent with our findings after repeating the analysis without the three left-handed participants and adding age and PASE as covariates (Supplementary Figure 3).

#### Laterality index (LI)

We tested for a difference in fNIRS response amplitude between corresponding channels in the left and right hemispheres to assess the lateralization of cortical activity. Table 2 shows the results of channel-wise significance testing for a main effect of hemisphere. The tests were two-tailed, allowing for the detection of both left- and right-lateralized activation. The laterality indices were calculated for all participants and were analyzed for any differences between the Okinawan and Dutch sample. No significant difference in lateralization between the samples from n-back test could be confirmed [t (73) = −0.4, *p* = 0.6]. LI was not significantly different between two groups for the phonemic verbal fluency task [t (59) = -1.13, *p* = 0.25]. LI was significantly different though for the semantic verbal fluency task, with a small mean difference of 0.36, pointing to less LI in Dutch older adults relative to Okinawan older adults.

**TABLE 2:** Laterality index (LI) results for the tasks in Groningen and Okinawa groups.

Group	Mean (SD)			Significance	Significance	Significance
				LI n-back	LI phonemic	LI semantic
	LI n-back	LI phonemic	LI semantic	Groningen vs. Okinawa		
Groningen	0.06 (0.61)	0.08 (0.59)	−0.09 (0.54)	t (73) = −0.4	t (59) = −1.13	t (59) = −2.18
Okinawa	0.13 (0.71)	0.28 (0.73)	0.27 (0.68)	(0.6)	(0.25)	(0.03)
Groningen	–	0.29 (0.7)	0.14 (0.8)	–	0.4	0.052
lateral						
channels						
Okinawa	–	0.45 (0.7)	0.54 (0.7)	–		
lateral						
channels						

Larger positive number indicates stronger left lateralization.

## Discussion

In this study we examined the difference in prefrontal brain activation between Dutch and Okinawan older adults during a verbal fluency task and a working memory task. Consistent with our prediction, our results revealed lower activation of the left lateral prefrontal cortex during verbal fluency and of the right lateral prefrontal cortex during working memory processing in the Okinawan as compared to the Dutch older adult participants. We suggest that Okinawan older adults may need less executive processing resources to perform the task, i.e., their brains may work more efficiently in this regard, closer to a youth-like pattern. While we caution our conclusion since we did not directly measure cognitive efficiency, a meta-analysis has shown that older adults hyper-activate the frontoparietal network during working memory, as compared to younger adults (Li et al., 2015). Consistent with this, a recent study reported that cognitive reserve (a construct based on factors that protect against cognitive decline) was associated with smaller increases in fNIRS-derived HbO2 in prefrontal areas from single tasks to dual-task walking in older adult participants (Holtzer et al., 2021). On the other hand. Lee et al. (2024) investigated brain activation during a visual memory span task in older adults with varying degrees of cognitive decline. They observed lower HbO levels specifically in participants with the amnestic type of mild cognitive impairment (aMCI) compared to participants with non amnestic types of MCI or older adults with normal cognition (NC). This pattern was also observed in NC individuals who reported memory problems.

We also observed other differences in brain activation between the two groups, which we did not predict. During the verbal fluency task, the Okinawa group had more activation in the more medial areas of the frontal gyrus as compared to the Groningen group. During the n-back working memory task, the Okinawa group showed more activation in anterior areas of the frontal cortex. Thus, there was no general reduction in brain activation in the Okinawa group versus Groningen group, but it was limited to specific task-relevant areas (that have also been shown to be task-relevant in prior studies). These other differences in activation may be related to different strategy use, which may be studied in more detail in future investigations.

Verbal fluency is a commonly used neuropsychological task that engages not only language processing, but also executive functioning such as monitoring (to keep track of which words were named) and inhibition (to avoid repeating the same words) (Birn et al., 2010, Lezak et al., 2004). Previous fMRI studies found that verbal fluency activates consistently and most robustly inferior and middle frontal gyri mainly in the left but also in the right hemisphere, in addition to the anterior cingulate, bilateral insulae and left superior frontal gyri (Birn et al., 2010, Indefrey and Levelt, 2000, Schlösser et al., 1998, Wagner et al., 2014, Weiss et al., 2003) (more in Supplementary materials). Additionally, fNIRS studies that measured frontal cortical HbO levels found robust activation of same brain regions (Herrmann et al., 2008, Kakimoto et al., 2009, Schecklmann et al., 2008). This confirms involvement of the inferior and middle frontal gyri, that were measured by the lateral channels of our optode montage, as most relevant for this task. Among these channels, we found higher levels of HbO in the Groningen group as compared to older adults from Okinawa. Thus, in the area relevant for the verbal fluency task, the Okinawa group showed, as predicted by our hypothesis, less activation. This is consistent with the idea that better preserved cognition in older adults from Okinawa (Willcox et al., 2007) is subserved by efficient brain functioning. That is, aging and cognitive decline have been associated with decline in metabolism and vascular function that affect brain structure and function (Chételat et al., 2013, Song et al., 2013). In order to preserve normal cognitive functioning compensatory mechanisms are needed (Hillary et al., 2015). This can take the form of reorganization of brain activation in order to partly and temporarily compensate for aging and neurodegenerative processes (Eyler et al., 2011, Stern, 2012). Such adaptations could manifest in several ways, for example by increased brain activation (Heinzel et al., 2013), or reassignment of involved brain areas which could lead to alteration in lateralization (Yeung et al., 2016). The latter was proposed as The Hemispheric Asymmetry Reduction in OLDer adults hypothesis (HAROLD) by Cabeza (2002). We will discuss lateralization in more detail after discussing the activation differences for the working memory task first.

For working memory, we selected a non-verbal n-bask task with objects as it is more culturally neutral and does not depend on language differences. fNIRS was previously used for measurement of frontolateral brain activity in healthy participants (Fishburn et al., 2014) and findings suggest that it can be successfully used to robustly quantify and classify mental workload (Herff et al., 2014). Herff et al. (2014) showed, that the activation in the lateral frontal gyrus increases with increasing load. Consistent with our hypothesis, we found less activation of the right lateral prefrontal cortex during working memory processing in the Okinawan as compared to the Dutch older adult participants. Studies using brain stimulation with rTMS have shown the right (and not the left) DLPFC to be involved in visual working memory, supporting the relevance of this region (Fried et al., 2014). Vermeij et al. (2017) investigated hemodynamic response in older adults and showed that a strength of response is related to working memory load. They concluded that a “youth-like” prefrontal activation pattern at older age may be associated with better behavioral outcome and cognitive plasticity.

Regarding hemispheric asymmetry differences, we observed significantly lower lateralization during category verbal fluency in Groningen group as compared to Okinawa group, though we did not find lateralization differences for letter fluency. Stronger lateralization for the Okinawan participants was predicted from our hypothesis of a more “youth-like” pattern. Results from the n-back task were inconsistent with our hypothesis, as no differences in LI were apparent. Lateralization of frontal areas in non-verbal tasks is not always observed (Mencarelli et al., 2019), however, even though a study with a visuospatial task did report more pronounced activation of the right hemisphere (Sanefuji et al., 2007). Lack of functional lateralization in brain activation does not mean that one hemisphere does not contribute more to the task than the other, as brain activation measures are correlational. Notably, previous findings with fNIRS show both decreased activation (Henry et al., 2004) and lower lateralization in people with diminished cognition (Weiss et al., 2004, Yeung et al., 2016). Both our groups are older adults, and for both the lateralization seems diminished which is consistent with the theory of age-related cortical reorganization (Heinzel et al., 2015).

### Limitations

The following limitations of this study should be mentioned. First, direct comparison of performance levels on the tasks is not straightforward, as the standard versions of the tests that are used in each country are different (see Supplementary materials). Most importantly, all participants fall within the normal range given the available normative values for that country. Education was also difficult to match as the standard scale used for education for Dutch participants is the Verhage scale (Verhage, 1964), which cannot be applied to the Japanese educational system. Also years of education are not applicable, as for the same education level there is different number of years necessary in Japan and Netherlands (Nuffic, 2015). However, classification of educational level in three categories (high, medium and low) revealed that the two groups did not differ for level of education. We recommend use of such classification to aid comparison (details in Supplementary materials, Supplementary Figure 1). A further limitation regarding the cross-linguistic comparisons of the letter condition in verbal fluency tasks concerns the structural differences between Japanese and Dutch. Japanese relies on moras—the smallest phonological units that influence timing and rhythm in speech—rather than individual letters or syllables as in Dutch (Stanlaw, 2004). In mora-based letter fluency tasks, the strong correspondence between moras and *kana* characters imposes a more rigid search strategy compared to the more flexible first-letter retrieval in Dutch. As a result, the number of accessible words may differ across languages, making direct comparison of task difficulty problematic.

These linguistic distinctions can also affect neural activation patterns. In Japanese speakers, the mora-letter fluency task not only activates the frontotemporal cortices—regions typically involved in semantic fluency—but also engages additional areas such as the middle frontal gyrus and the supramarginal gyrus in the left hemisphere. Nevertheless, the frontal regions targeted in this study are consistently associated with verbal fluency processing across languages, sex, and cultures (Dan et al., 2013; Herrmann et al., 2003; Ren et al., 2024), suggesting some degree of neural commonality.

Despite these structural and neural differences, it is reasonable to assume that participants in both language groups engaged in the core cognitive process of lexical retrieval during the task. Ardila (2020), in a cross-linguistic study of verbal fluency across 15 languages, reported remarkably similar performance, although his findings were limited to category fluency (e.g., animals). Letter fluency may be more sensitive to linguistic variation. Ardila also emphasized a cultural factor often overlooked in such studies: the degree of effort invested in test performance, which varies depending on how much value a culture places on psychometric assessment. How such cultural and linguistic factors translate into differences in brain activation remains an open question.

Similarly, Pekkala et al. (2009) found no significant differences in semantic verbal fluency performance between American English-speaking and Finnish-speaking elderly participants, suggesting that culture and language may not substantially affect SVF outcomes in healthy older adults. However, the influence of language structure and cultural attitudes on letter fluency—and their neural correlates—merits further investigation.

Kempler et al. (1998) studied semantic verbal fluency (animals) performance in a group of 317 healthy participants between 54 and 99 years of age. These included Chinese, Hispanic, and Vietnamese immigrants, as well as White and African American English speakers, who named as many animals as possible in 1 min, in their native language. Lower age and higher education were associated with better performance. Language background was also relevant: The Vietnamese produced the most animal names and the Spanish speakers produced the fewest. The authors attribute this to the fact that Vietnamese animal names are short (predominantly 1 syllable) while the Spanish animal names are longer than in the other languages (2 and 3 syllables per word). To conclude, language and culture may have some influence, and cannot be discarded as a contributing to the performance of participants in our study.

This brings us to another limitation of our study: we did not investigate possible causes of the brain activation differences between groups. This is beyond the scope of the current study but should be addressed in future research. Lifestyle differences, such as dietary intake, social connectedness and exercise, are examples of variables that have been suggested to underlie healthy aging in older adults from Okinawa (Santacroce et al., 2024; Poulain and Herm, 2024). Diet and exercise have been shown to improve brain health (Key and Szabo-Reed, 2023), which in turn could delay age-related decline.

## Conclusion

In conclusion, our results support the hypothesis of reduced task-related frontal activation during verbal fluency and non-verbal working memory in older adults from Okinawa as compared to Dutch older adults. This could be hypothesized to be due to more efficient neural processing, though there were other differences between the two groups, that may be suggestive of different cognitive strategy use. Our study should be regarded as a first step into elucidating the neural basis of healthy cognitive aging that is characteristic of older adults people in Okinawa and for healthy brain aging in general. Elucidation of the specific ingredients (e.g., lifestyle characteristics) that contribute to healthy brain aging will potentially aid the development of prevention programs for cognitive decline in the elderly.

## Data Availability

The raw data supporting the conclusions of this article will be made available by the authors, upon request.

## Footnotes

1
www.nirx.net
