# Supplementary material for: Difference between Okinawan and Dutch older adults in prefrontal brain activation
Source: Front Aging Neurosci. 2025 Jul 31;17:1454068. doi: 10.3389/fnagi.2025.1454068 (PMC12350274; doi:10.3389/fnagi.2025.1454068)
Supplement: Supplementary file 1 [file Data_Sheet_1.pdf]

Supplementary materials to

Difference between Okinawa and Dutch older adults in prefrontal brain activation

Branislava Ćurčić-Blake, Yuko Futemma, Donald Craig Willcox, Parisa Esmaeili Tazangi, Nido Dipo Wardana, Yukihiko Ueda, André Aleman

Methods

NIRS acquisition

Table S1 Source-Detector Locations and Associated Anatomical Regions

	Left hemisphere		Anatomical regions[a]	Right hemisphere	
	Optode	10/20 System		Optode	10/20 System
Sources	S1	Fp1	Superior frontal gyrus (rostral)	S5	Fp2
	S2	F7	Inferior frontal gyrus (rostral)	S6	F8
	S3	AFF5h	-	S7	AFF6h
	S4	Between AF3 and Fp1	-	S8	Between AF4 and Fp2
Detectors	D1	AF7	Middle frontal gyrus	D5	AF8
	D2	F5	Middle frontal gyrus	D6	F6
	D3	AF3	Superior frontal gyrus	D7	AF4
	D4	Between F7 and F5	-	D8	Between F8 and F6

Note. [a] Source: Koessler and associates (2009); Homan, Herman, and Purdy (1987).

Table S2. Source-Detector locations

Channel	Source	Detector	Distance [mm]
1	1	1	30.39
2	1	3	31.59
3	2	1	30.49
4	2	2	30.76
5	2	4	15.40
6	3	1	20.32
7	3	2	33.15
8	3	3	20.17
9	3	4	36.22
10	4	1	32.93
11	4	3	14.11
12	5	5	29.82
13	5	7	31.42
14	6	5	30.00
15	6	6	30.55
16	6	8	15.30
17	7	5	20.10
18	7	6	32.62
19	7	7	19.96
20	7	8	35.66
21	8	5	31.33
22	8	7	14.69

TableS2. Channels between the sources and detectors. The left column lists the channel number. The second and the third columns from the left list the source and detectors respectively. The channel refers to the measurements between the respective source and detector. The fourth column lists distances between sources and detectors in mm.

Educational level adjustment

Number of years and completed level of education were self-reported by participants. Education is an important factor that can affect cognitive functions. There are differences in Dutch and Japanese educational system. For instance, Dutch primary education (8-year period) is intended for children in the age group of 4 to 12 and is compulsory for children from the age of 5. Then, they need to spend 5 years in high school (in total 13 years) to receive a Dutch HAVO diploma or high school certificate. However, the Japanese compulsory education is comprised of primary education (6-year period) from

6 years old and lower secondary education (3-year period), lasting a total of 9 years. They need to spend another three years in high school, which sums up to twelve years of education in order to receive a Japanese High School Certificate of Graduation.

We adjusted Japanese educational system to Dutch educational system in order to match the educational level in both groups. This was done according to the different reports by Nuffic (8), a Dutch organization for the internationalization of education, and the Dutch Qualification Framework (NLQF) (Table S3). To classify the level of education system, the Dutch Verhage scale was used (9). The seven categories were merged into three ordinal categories: low educational level (Verhage 1 until 4), middle educational level (Verhage 5), and high educational level (Verhage 6 and 7; Table S3). Table S4 shows the numbers for each group per educational category.

Table S3. Description of educational levels

Level		Verhage category[a]	NLQF[b]/EQF[c]	The Netherlands	Japan
Low	1	less than primary school/primary school not finished	Entry Level	Basic Education (Primary)	Primary School
	2	Finished primary school	1	MBO1	Junior High school
	3	Completed primary school and further education less than 2 years	2	MBO 2/ vmbo kb, gl, tl	
	4	lower than MULO/MAVO level, e.g. LTS, LEAO, LHNO	3	MBO 3	Upper Secondary Vocational school
Middle	5	MULO/MAVO/MEAO diploma	4	Havo/ Vwo/ Vavo-Havo	High School certificate
High	6	HAVO/VWO/HEAO/HBS/HBO diploma	5	Associate Degree	Diploma at a professional level/Junior college/College of technology
	7	university diploma	6	Bachelor Degree	Advanced Diploma/Bachelor's degree
			7	Master Degree	Master Degree
			8	Doctorate	Doctorate

[a] Adapted from Verhage (1964). [b] Dutch Qualification Framework. [c] European Qualifications Framework

Table S4. Participants' distribution in each educational level

Tests	Total No.		Educational Level (n)					
	Groningen	Okinawa	High		Middle		Low	
			Groningen	Okinawa	Groningen	Okinawa	Groningen	Okinawa
**Verbal Fluency**	24	37	23	32	0	0	1	5
**n-back**	38	37	36	32	1	0	1	5

Conversion from optical density to Hb and HbO levels

The absorption data were converted into concentration data using the modified Beer-Lambert Law (mBLL): $OD_\lambda = \left(\varepsilon_{HbO_2}^{\lambda}[HbO_2] + \varepsilon_{HbR}^{\lambda}[HbR]\right) \cdot DPF \cdot d + G$ (Zhao et al., 2017). Here, OD denotes optical density or absorption, λ the wavelength of the light, ε denotes the extinction coefficient, the d in the formula denotes the distance between the source and the detector, while G represents loss of light intensity due to scattering. We used the absorption spectra provided by Gratzer and associates (Gratzer

and Kollias, 1999) to determine the ε of HbO and Hb for each wavelength. DPF is the differential pathlength factor and the current study used the DPFs provided by Essenpreis and colleague (Essenpreis et al., 1993). Eventually, the changes of concentration values were calculated.

Additional measures

A number of tests and questionnaires were used to characterize the groups in terms of cognitive status, social functioning and physical activity. The neuropsychological tests include: (a) Digit Span is a subtest included in both the Wechsler Memory Scale-III (WSM-III) and the Wechsler Adult Intelligence Scale (WAIS) and consists of two versions (10). The Digits Forward (Forwards) is considered as a simple memory span test, while the Digits Backward (Backwards) is a more complex test with an executive component (11); (b) Symbol Digit Modalities Test (SDMT) was originally developed to identify neuropsychological impairments and may assess divided attention, visual scanning, and motor speed (12,13); (c) Trail Making Test (TMT) consists of two parts where part A (TMT-A) assesses attention and speed, while part B (TMT-B) also involves divided attention and mental flexibility ; (d) Stroop Test was initially developed by Stroop (14) and is a measure of selective attention and cognitive flexibility; and (e) Mini Mental State Examination (MMSE) was developed by Folstein, and McHugh (1975) and has been a popular measure to screen cognitive impairments especially in older adults (13,15). In addition, we administered other screening measures, including (f) Edinburgh Handedness Inventory is a 10-item questionnaire assessing handedness which was developed by Oldfield (16); (g) Social Functioning Scale (SFS) assesses seven areas that are crucial for social competence, namely social engagement/withdrawal, interpersonal behavior, prosocial activities, recreation, competence for independence, the performance of independence, and employment or occupation (17); and (h) Physical Activity Scale for the Elderly (PASE) assesses physical activities commonly engaged in by older persons and can be administered in person or via telecommunication (18).

There are some differences in two standard tests administered to the Dutch and Okinawan populations. The Trail Making Test is adapted to each population: the version normalized for the Japanese population is laid out horizontally, whereas the Dutch version is vertical (see Fig. S1a and b). Similarly, the Stroop Test also differs, with the Dutch version using the Latin alphabet and the Japanese version using Japanese characters (see Fig. S1c and d).

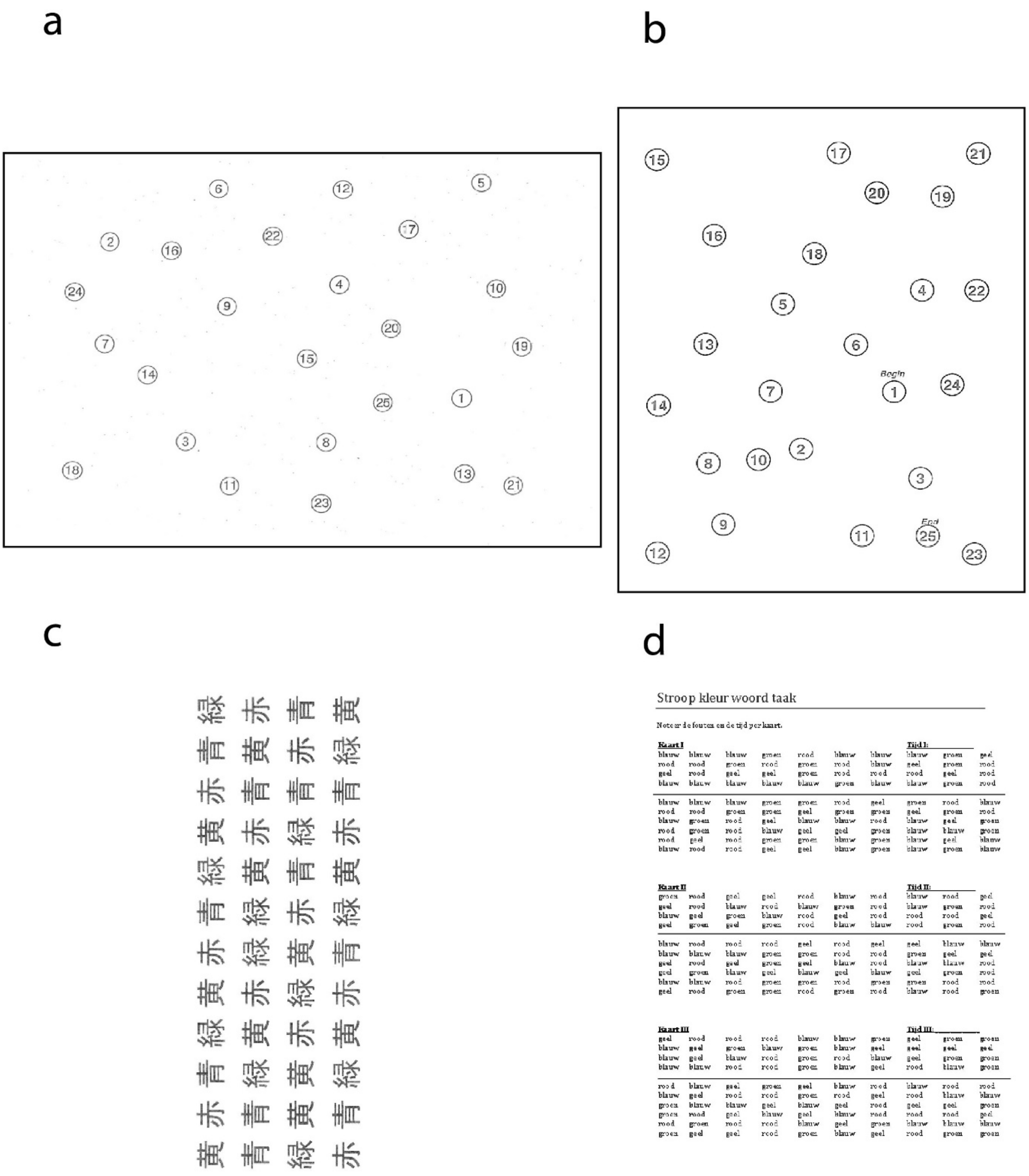

Figure S1 Examples of cognitive tests: a. TMT in Japanese population. b. TMT in Dutch population. c. Stroop test in Japanese population. d. Stroop test in Dutch population.

Results

Demographics

Table S5 summarizes all the demographics and behavioral data. The two groups were matched for sex (Table S5). There was difference in age of 2.9 years which was statistically significant. However, all participant were between 64 and 80 (Groningen participants were up to 77 years old), thus belonging

to the same age range. Educational level (according to the classification in Table S3) did not differ significantly between groups. In addition, The groups did not differ in cognitive and social functioning measures (i.e., MMSE and SFS) but, as expected, there was a difference in the physical activities as measured by PASE.

Regarding the screening measures, test performance was generally comparable between the two groups. Nevertheless, it is essential to note that some test performances are not comparable due to different standard versions of the screening tests used. The Groningen site utilized the forward digit span test from the WAIS-R which starts with a 3-digit number, while the Okinawan site used another version from the WAIS-III which starts with a 2-digit number. For the TMT, the Japanese version was administered on a horizontal A4 paper, while the Dutch version was presented on a vertical A4 paper. The two versions also differ in terms of the constellation pattern of numbers and letters. Additionally, the beginning and end points were pointed out to Dutch participants, while this procedure was missing in the Okinawan group. Lastly, the Japanese version of the Stroop test has only 48 items in each part, while the Dutch one has 100 items.

Performance on the verbal fluency and n-back tasks

The verbal fluency results showed that participants named different on number of words all four conditions (Table S5). The Okinawan elderly named (M=7.18, SD=3.07) words with the letter "A", while Dutch elderly named (M=11.62, SD=3.22). Similarly, in the "K" condition, the Okinawan elderly named (M=9.29, SD=3.14) words while the Dutch elderly named (M=14.58, SD=4.89). Furthermore, in the semantic condition "animals", the Okinawan elderly named (M=15.25, SD=2.97) and the Dutch elderly (M=21.79, SD=5.26),. And lastly, in the semantic condition "occupations", the Okinawan elderly named (M=10.64, SD=2.64) words which is less compared to the Dutch elderly (M=18.83, SD=5.89).

However, as explained in more detail in the Discussion, the Dutch participants had a clear "advantage" in the phonemic conditions. Because of language differences, they had a bigger pool of words to choose from compared to the Okinawan participants. Furthermore, normative data show that the mean words recalled by the Okinawan group fall within the normal age range for both the letter and category condition (19). Normative data for the Dutch population shows that the values of the categories "animals" and "occupations" fall within a normal range as well (20). Unfortunately, there is no normative data for the Dutch population for the letter conditions "A" and "K" for duration of 1 minute (the norms are given for 3 letters consecutively -total duration of 3 minutes). Thus, the performance on the VF task of both groups falls within norms.

Table S5a Verbal Fluency Task Results in Groningen and Okinawa Groups

Variable	Mean (SD)	
	Groningen (n=24)	Okinawa (n=37)
Phonemic Letter "A"	11.62 (3.22)	7.18 (3.07)

Phonemic Letter “K”	14.58 (4.89)	9.29 (3.14)
Semantic “Animals”	21.79 (5.26)	15.25 (2.97)
Semantic “Occupations”	18.83 (5.89)	10.64 (2.64)

Table S5b Normalized values for Verbal Fluency Category Task Results in Groningen and Okinawa Groups

Variable	Z (SD)				*t*-value (p-value)
	**Groningen (n=24)**	**Normalized Mean (SD)**	**Okinawa (n=37)**	**Normalized Mean (SD)**	
Semantic “Animals”	-0.47 (0.90)	24 (6)	0.68 (0.70)	12.4 (4.2)	6.13 (<0.001)
Semantic “Occupations”	0.11 (0.83))	17 (5)	0.85 (0.77))	7.8 (3.4)	3.96 (<0.001)

Table S5a and b. Descriptive statistics for performance on the VF per group are included. The left column lists the VF subtests. **S5a**. The second and third columns from the left show the mean and standard deviation per subtest. In **S5b**, the second and third columns show the average z-values of the variables across the group, with standard deviations in brackets. A non-parametric test (Mann–Whitney U test) was used to assess group differences for the sTMT and Stroop tasks. The third and fourth columns from the left show the normalized mean and standard deviation used to calculate the corresponding z-values. Normalized values were taken from Schmand et al. (2012) [20] for the Dutch population and from Ito et al. (2006) [33] for the Japanese population.

The result of n-back tests is shown in Table S6. The two groups had same accuracy. They differed in terms of proportions of true negative (U=303.5, *p*-value<0.001) and false positive detection (U=303.5, *p*-value<0.001) with Dutch participants demonstrated better performance. Although significant, these differences only translate to roughly 2 to 3 trials which are not practically meaningful. Further, the Dutch group also completed more trials on average than the Okinawa counterpart (t (73)=3.12, *p*-value<0.01).

Table S6. N-back task performance in Groningen and Okinawa groups

Variable	Median (SD)		Significance
	Groningen (n=38)	Okinawa (n=37)	Groningen vs. Okinawa
Accuracy	0.79 (0.09)	0.74 (0.14)	U= 559 (0.12)
True negative	0.91 (0.06)	0.81 (0.11)	U=303.5 (p<0.001)
False positive	0.08 (0.06)	0.18 (0.11)	U=303.5 (p<0.001)
Miss	0.2 (0.09)	0.25 (0.14)	U=559 (0.12)
Number of trials	25.15 (3.02)	22.82 (3.41)	t (73)=3.12 (0.003)

HbO levels after including handedness, age and PASE:

Supplementary Figures:

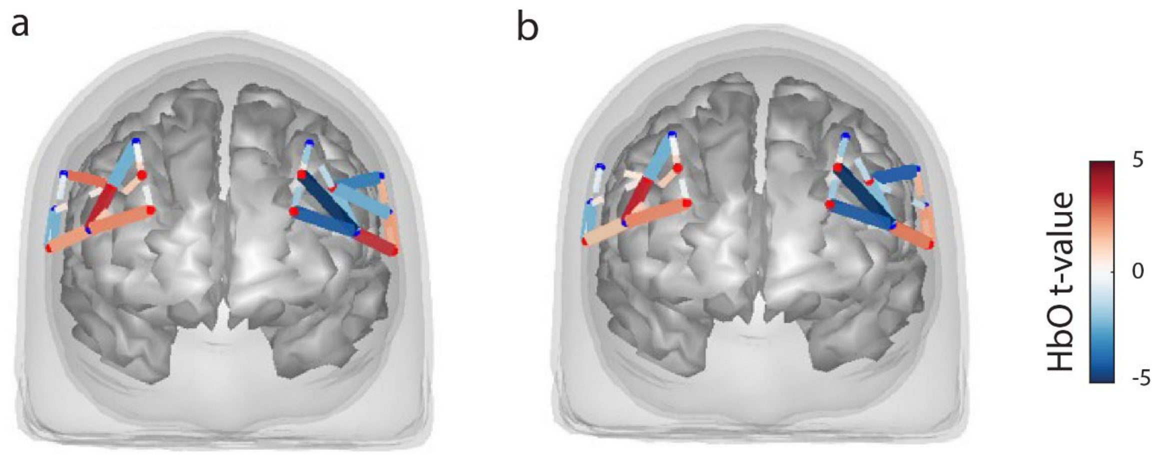

Figure S2 a. Verbal Fluency Level Contrasts for HbO after the correction for education – three left-handed participants were excluded. Only significant channels (p_{FDR}<0.05) are shown. For HbO contrasts, positive t-values (red) correspond to relatively larger activity in the Groningen group as compared to Okinawa group, and negative t-values (blue) correspond to larger activity for the Okinawa group. **b.** Same as a. after correction for PASE and age.

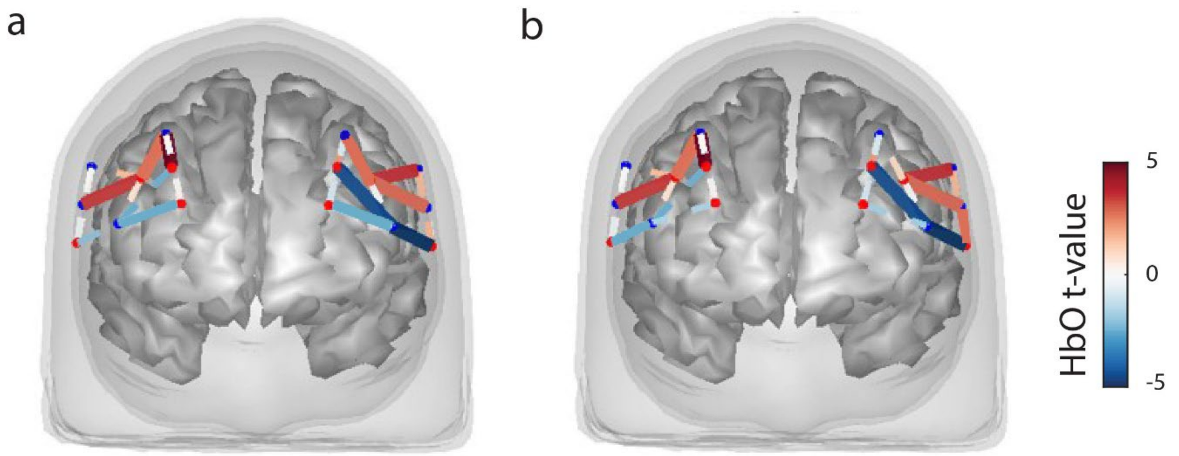

Figure S3 a. N-back level contrasts for HbO after the correction for education – three left-handed participants were excluded. Only significant channels (p_{FDR}<0.05) are shown. For HbO contrasts, positive t-values (red) correspond to relatively larger activity in the Groningen group as compared to Okinawa group, and negative t-values (blue) correspond to larger activity for the Okinawa group. **b.** Same as a. after correction for PASE and age.

Discussion

Verbal fluency is a commonly used neuropsychological task that engages not only language processing, but also executive functioning such as monitoring (to keep track of which words were named) and inhibition (to avoid repeating the same words) (7,21). Previous fMRI studies found that verbal fluency activates consistently and most robustly inferior and middle frontal gyri mainly in the left but also in the right hemisphere, in addition to the anterior cingulate, bilateral insulae and left superior frontal gyri (21-25). Additionally, fNIRS studies that measured frontal cortical HbO levels found robust activation of same brain regions (26-28).

Table S7 z-values for descriptive statistics for the samples, including cognitive test performance

Variable	Z-value (SD)		Normalized Mean (SD) or range		Significance t values* (*p*-value)
	Groningen (n=38)	Okinawa (n=37)	Groningen normalized	Okinawa normalized	Groningen vs. Okinawa
Digit Span					
Forward[a]	-0.46 (1.16)	1.6 (0.89)	7.1 (1.8)	5.85 (1.82)	t (73)=- 8.91 (<0.001)
Backward	1.31 (1.52)	0.59 (1,53)	4.6 (1.4)	4.83 (1.19)	t (73)=-2.05 (0.022)
TMT					
TMT-A[a]	-1.46 (1.3)	7.45 (3.68)	40 (6)	46.5 (10.5)	U=0 (<0.001)
TMT-B[a]	-0.97 (1.49)	2.47 (2.17)	86 (16)	88.8 (24.7)	U=77 (<0.001)
Stroop test					
Stroop I – time[a]	3.27 (4.22)	1.18 (1.25)	41-43	32-37[d]	U=1271 (<0.001)
Stroop II – time[a]	12.35 (3.52)	-3.32 (1.63)	56-59	41-45 [d]	U=1406 (<0.001)
Stroop III – time[a]	-0.39 (2.75)	-2.37 (1.46)	94-101	73-84 [d]	U=1042 (<0.001)

Table 1. Descriptive statistics for the groups. The left column lists the demographic variables and cognitive test performance. The second and third columns from the left show the average z-values of the variables across the group, with standard deviations in brackets. A non-parametric test (Mann–Whitney U test) was used to assess group differences for the sTMT and Stroop tasks. TMT = Trail-Making Task. The third and fourth columns from the left show the normalized mean and standard deviation used to calculate the corresponding z-values. The normalized values for Digit Span were

based on Beker et al. (2018) [29] for the Dutch population and Dodge et al. (2008) [30] for the Japanese population. For the Dutch population, the TMT and Stroop test values were based on Schmand et al. (2012) [20]. For the Japanese population, the TMT normalized values were based on Harada et al. (2006) [31], and the Stroop task values were based on Tominaga (2008) [32].

References

(1) Ikezawa K, Iwase M, Ishii R, Azechi M, Canuet L, Ohi K, et al. Impaired regional hemodynamic response in schizophrenia during multiple prefrontal activation tasks: a two-channel near-infrared spectroscopy study. Schizophr Res 2009 Mar;108(1-3):93-103.

(2) Santosa H, Zhai X, Fishburn F, Huppert T. The NIRS Brain AnalyzIR Toolbox. Algorithms 2018;11(5).

(3) Zhang S, Zheng Y, Wang D, Wang L, Ma J, Zhang J, et al. Application of a common spatial pattern-based algorithm for an fNIRS-based motor imagery brain-computer interface. Neurosci Lett 2017;655:35-40.

(4) Zhao Y, Qiu L, Sun Y, Huang C, Li T. Optimal hemoglobin extinction coefficient data set for near-infrared spectroscopy. Biomed Opt Express 2017 Oct 23;8(11):5151-5159.

(5) Gratzer WB, Kollias N. Tabulated molar extinction coefficient for hemoglobin in water. Wellman Laboratories, Harvard Medical School, Boston 1999;5:50-161.

(6) Essenpreis M, Elwell CE, Cope M, van der Zee P, Arridge SR, Delpy DT. Spectral dependence of temporal point spread functions in human tissues. Appl Opt 1993 Feb 1;32(4):418-425.

(7) Lezak MD, Howieson DB, Loring DW. Neuropsychological Assessment (4th ed.). New York: Oxford University Press; 2004.

(8) Education System Japan. 2015.

(9) Verhage F. Intelligence and age: Survey in Dutch twelve to seventy year olds, In Dutch: Intelligentie en leeftijd; onderzoek bij Nederlanders van twaalf tot zevenenzeventig jaar. Assen,: Van Gorcum,; 1964.

(10) Griffin PT, Heffernan A. Digit Span, Forward and Backward: Separate and Unequal Components of the WAIS Digit Span. Percept Mot Skills 1983;56(1):335-338.

(11) Wilde NJ, Strauss E, Tulsky DS. Memory Span on the Wechsler Scales. 2004;26(4):539-549.

(12) Sheridan LK, Fitzgerald HE, Adams KM, Nigg JT, Martel MM, Puttler LI, et al. Normative Symbol Digit Modalities Test performance in a community-based sample. Arch Clin Neuropsychol 2006;21(1):23-28.

(13) Strauss E, Sherman EMS, Spreen O. A Compendium of Neuropsychological Tests: Administration, Norms, and Commentary. : Oxford University Press; 2006.

(14) Stroop JR. Studies of interference in serial verbal reactions. Journal of Experimental Psychology 1935;18(6):643-662.

(15) Folstein MF, Folstein SE, McHugh PR. "Mini-mental state". A practical method for grading the cognitive state of patients for the clinician. J Psychiatr Res 1975 Nov;12(3):189-198.

(16) Oldfield RC. The assessment and analysis of handedness: The Edinburgh inventory. Neuropsychologia 1971;9(1):97-113.

(17) Birchwood M, Smith J, Cochrane R, Wetton S, Copestake S. The Social Functioning Scale. The development and validation of a new scale of social adjustment for use in family intervention programmes with schizophrenic patients. Br J Psychiatry 1990;157(6):853.

(18) Washburn RA, Smith KW, Jette AM, Janney CA. The physical activity scale for the elderly (PASE): Development and evaluation. Journal of Clinical Epidemiology 1993;46(2):153-162.

(19) E. Itou. Neuropsychological studies of verbal fluency test. In: Doctoral thesis, editor. Neuropsychological studies of verbal fluency test Nagoya, Japan: Nagoya University Graduate School of Environmental Studies.; 2006.

(20) Schmand B, Houx P, de Koning I. Normen van psychologische tests voor gebruik in de klinische neuropsychologie. Excelbestand met bijbehorende toelichting. 2012; Available at: www.psynip.nl/website/sectoren-en-secties/sector-gezondheidszorg/neuropsychologie.

(21) Birn RM, Kenworthy L, Case L, Caravella R, Jones TB, Bandettini PA, et al. Neural systems supporting lexical search guided by letter and semantic category cues: A self-paced overt response fMRI study of verbal fluency. Neuroimage 2010;49(1):1099-1107.

(22) Wagner S, Sebastian A, Lieb K, Tüscher O, Tadić A. A coordinate-based ALE functional MRI meta-analysis of brain activation during verbal fluency tasks in healthy control subjects. BMC Neuroscience 2014;15(1):19.

(23) Indefrey P, Levelt WJM. The neural correlates of language production. In: Gazzinga MS, editor. The new cognitive neurosciences. 2nd ed.: Cambridge, MA: MIT Press; 2000. p. 845-865.

(24) Schlösser R, Hutchinson M, Joseffer S, Rusinek H, Saarimaki A, Stevenson J, et al. Functional magnetic resonance imaging of human brain activity in a verbal fluency task. J Neurol Neurosurg Psychiatr 1998;64(4):492.

(25) Weiss EM, Siedentopf C, Hofer A, Deisenhammer EA, Hoptman MJ, Kremser C, et al. Brain activation pattern during a verbal fluency test in healthy male and female volunteers: a functional magnetic resonance imaging study. Neurosci Lett 2003;352(3):191-194.

(26) Kakimoto Y, Nishimura Y, Hara N, Okada M, Tanii H, Okazaki Y. Intrasubject reproducibility of prefrontal cortex activities during a verbal fluency task over two repeated sessions using multi-channel near-infrared spectroscopy. Psychiatry Clin Neurosci 2009;63(4):491-499.

(27) Herrmann MJ, Langer JBM, Jacob C, Ehlis A, Fallgatter AJ. Reduced Prefrontal Oxygenation in Alzheimer Disease During Verbal Fluency Tasks. The American Journal of Geriatric Psychiatry 2008;16(2):125-135.

(28) Schecklmann M, Ehlis A, Plichta MM, Fallgatter AJ. Functional near-infrared spectroscopy: A long-term reliable tool for measuring brain activity during verbal fluency. Neuroimage 2008;43(1):147-155.

(29) Beker, N., Sikkes, S.A.M., Hulsman, M., Schmand, B., Scheltens, P., Holstege, H., 2018. Neuropsychological Test Performance of Cognitively Healthy Centenarians: Normative Data From the Dutch 100-Plus Study. J American Geriatrics Society 67, 759.

(30) Dodge, H.H., Kita, Y., Takechi, H., Hayakawa, T., Ganguli, M., Ueshima, H., Hall, W., Ferrucci, L., 2009. Healthy Cognitive Aging and Leisure Activities Among the Oldest Old in Japan: Takashima Study.

(31) Harada, H., Notoya, M., Nakanishi, M., Fujiwara, N., Inoue, K., 2006. Effects of age and years of education on neuropsychological data of Japanese healthy elderly persons. Higher Brain Function Research 26, 16–24.

(32) Tominaga, D., 2008. The standardization of the Ryudai's Stroop Test（ＲＳＴ）. Bulletin of Faculty of Education 72, 27–32.

(33) Ito E., 2006. Neuropsychological studies of verbal fluency tests.
